# Unrestricted Kinematic Alignment Is a Feasible Strategy for Lateral Compartment Osteoarthritis: Short-Term Outcomes of a Contralateral Knee-Referenced Approach

**DOI:** 10.3390/jcm15041606

**Published:** 2026-02-19

**Authors:** Yong Deok Kim, Sueen Sohn, Se Heon Lee, Nicole Cho, In Jun Koh

**Affiliations:** 1 Joint Replacement Center, Eunpyeong St. Mary’s Hospital, Seoul 03312, Republic of Korea; seraph622@naver.com (Y.D.K.); dltpgis202@naver.com (S.H.L.); 2 Department of Orthopaedic Surgery, College of Medicine, The Catholic University of Korea, Seoul 06591, Republic of Korea; 3 Department of Orthopaedic Surgery, Inje University Sanggye Paik Hospital, College of Medicine, Inje University, Seoul 01757, Republic of Korea; osdocsse@gmail.com; 4 Hackensack Meridian School of Medicine, 123 Metro Blvd, Nutley, NJ 07100, USA; nicole.cho@hmhn.org

**Keywords:** lateral compartment osteoarthritis, coronal plane alignment, unrestricted kinematic alignment, personalized alignment, total knee arthroplasty

## Abstract

**Background/Objectives**: Although unrestricted kinematic alignment (uKA) has gained increasing acceptance in total knee arthroplasty (TKA), its application in knees with lateral compartment osteoarthritis (OA) remains a subject of debate due to concerns over postoperative gap imbalance and alignment outliers. The purpose of this study was to evaluate the surgical, radiographic, and clinical outcomes of contralateral non-OA knee–referenced, caliper-verified uKA in lateral compartment OA. **Methods**: This retrospective study included 40 patients with isolated lateral compartment OA who underwent primary TKA using contralateral non-OA knee–referenced, caliper-verified uKA. Surgical outcomes were assessed by measuring bone resection thicknesses of the distal femur, posterior femur, and proximal tibia, as well as extension and 90° flexion gaps. Radiographic outcomes included mechanical hip–knee–ankle angle, medial proximal tibial angle, lateral distal femoral angle, and Coronal Plane Alignment of the Knee (CPAK) classification. Patient-reported outcomes (PROs), including Pain VAS, EQ-5D, satisfaction, and Forgotten Joint Score, were assessed at a minimum follow-up of 2 years. **Results**: The resected osteochondral thickness was consistently greater on the medial side than on the lateral side, and all gap balances were well maintained, with a gap difference ≤ 2 mm observed in 95% of knees in full extension. Postoperatively, restoration to the same CPAK category was achieved in approximately 90% of cases. All PROs improved and reached levels comparable to those of the contralateral knee. **Conclusions**: In patients with lateral compartment OA, caliper-verified uKA may be appropriately applied when guided by a reliable anatomic reference, such as the contralateral non-OA knee. This strategy achieves stable soft-tissue balance, reliable coronal alignment restoration, and favorable clinical outcomes in carefully selected valgus knees undergoing TKA.

## 1. Introduction

With advances in total knee arthroplasty (TKA), alignment strategies have shifted from a systematic approach toward a personalized approach [1]. Among these strategies, unrestricted kinematic alignment (uKA) represents one of the most prominent patient-specific concepts, aiming to restore each patient’s native knee alignment, joint-line obliquity, and soft-tissue laxity [2]. Previous biomechanical and clinical studies have demonstrated that uKA can better reproduce native knee kinematics and may provide superior functional outcomes and patient satisfaction compared with mechanically aligned TKA [3,4,5,6,7]. However, most KA TKA studies have focused on medial compartment osteoarthritis (OA) in varus knees [2,8,9,10]. In contrast, lateral compartment OA is less prevalent and typically presents with valgus alignment, posing unique anatomical and biomechanical challenges [11].

Valgus knee deformity is characterized by complex structural and biomechanical alterations involving both osseous and soft-tissue components around the knee joint. These changes commonly include lateral femoral condylar hypoplasia, asymmetric wear of the distal and posterior femoral condyles, lateral compartment cartilage degeneration with subchondral remodeling, and relative laxity of the medial soft tissues. Such alterations often result in lateral compartment narrowing and widening of the medial joint space, accompanied by valgus angulation of the femur and tibia and rotational abnormalities of the lower limb. Additional structural abnormalities, such as posterolateral tibial plateau deficiency and patellofemoral joint changes, including lateral patellar subluxation, may further complicate joint mechanics. These synergistic anatomical and biomechanical features increase the technical difficulty of achieving appropriate alignment and soft-tissue balance during total knee arthroplasty in the valgus knee [12,13,14,15,16,17].

In lateral compartment OA, the application of uKA has raised several concerns. uKA may accentuate preexisting valgus deformity, potentially compromising implant longevity and alignment-related outcomes [18]. In addition, residual valgus alignment after TKA has been reported to be associated with worse patient satisfaction and clinical outcomes [19,20]. Moreover, valgus knees often exhibit medial soft-tissue laxity, raising concerns regarding postoperative gap imbalance and instability when alignment is restored toward the native phenotype [21]. Furthermore, there is also concern that KA in valgus knees may adversely affect patellofemoral (PF) biomechanics, leading to impaired PF function [22]. Moreover, most available studies include mixed alignment phenotypes or employ restricted kinematic alignment (rKA) or functional alignment (FA), which are frequently preferred strategies in valgus knees [23,24,25]. Therefore, data specifically evaluating contralateral-referenced, caliper-verified uKA in patients with isolated lateral compartment OA remain scarce.

The purpose of this study was to evaluate the outcomes of caliper-verified uKA using the contralateral non-OA knee as a reference in patients with lateral compartment OA. Specifically, we investigated: (1) surgical outcomes in terms of bone resection thickness and gap measurements; (2) radiographic outcomes focusing on restoration of coronal plane alignment of the knee; and (3) changes in PROs, including Pain VAS, satisfaction, EQ-5D, and Forgotten Joint Score (FJS). We hypothesized that, in valgus knees with lateral compartment OA, contralateral non-OA knee–referenced uKA would restore coronal alignment and PROs to levels comparable to those of the contralateral non-OA knee.

## 2. Materials and Methods

This retrospective study included patients who underwent caliper-verified uKA TKA at a single institution between January 2021 and January 2024. We included consecutive patients who met all of the following: (1) unilateral valgus deformity attributable to isolated lateral compartment OA with Kellgren-Lawrence (KL) grade 4; (2) contralateral knee KL ≤ 2 (constitutional reference limb); (3) primary TKA performed using a caliper-verified uKA technique; and (4) a minimum clinical and radiographic follow-up of 2 years. Patients with prior knee surgery, inflammatory arthritis, or bilateral end-stage OA were excluded. After applying these criteria, 40 patients were enrolled in the study. The patients comprised 32 women (80%) and 8 men, with a mean age of 71.4 ± 8.3 years. The mean body mass index was 24.8 ± 3.1 kg/m^2^, and the mean body weight and height were 61.1 ± 9.9 kg and 156.7 ± 7.1 cm, respectively (Table 1). This study was approved by our hospital’s institutional review board (PC24RISI0156).

**Figure 1:**
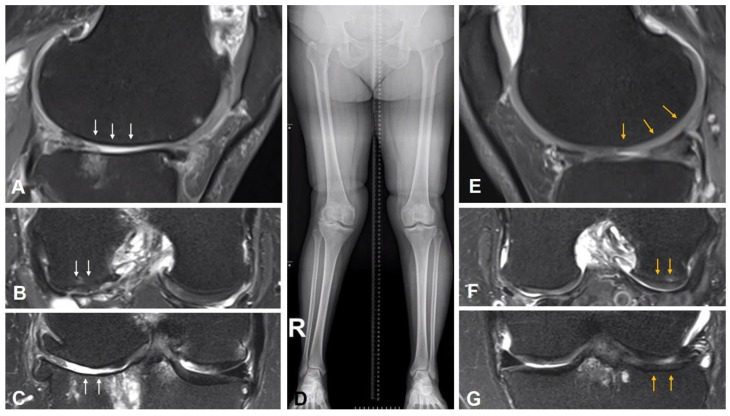
Representative imaging findings in lateral compartment OA and the contralateral non-OA knee. Preoperative MRI of the OA knee demonstrating lateral compartment cartilage degeneration, subchondral bone alteration (arrows) (**A**–**C**). Preoperative full-length standing anteroposterior radiograph showing coronal limb alignment (**D**). MRI of the contralateral non-OA knee demonstrating relatively preserved cartilage and subchondral bone compared with the OA side (arrows) (**E**–**G**). These images illustrate the rationale for using the contralateral knee as an anatomic reference for uKA.

**Figure 2:**
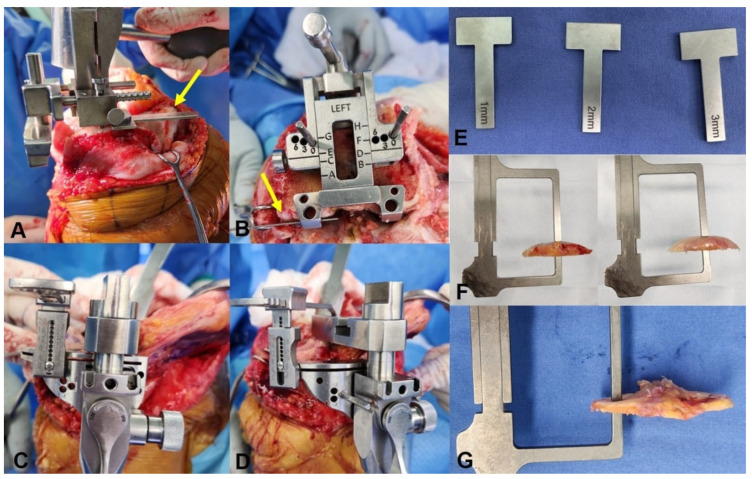
Caliper-verified surgical technique for contralateral knee–referenced uKA. Intraoperative photographs showing caliper-verified measurement of bone resection thickness during TKA using the uKA principle (**A**–**D**). Metal calibration bars with predefined thicknesses (1–3 mm) used for intraoperative calibration (**E; yellow arrows**). Representative resected bone specimens positioned within the measuring frame for verification of actual resection thickness (**F**,**G**).

**Figure 3:**
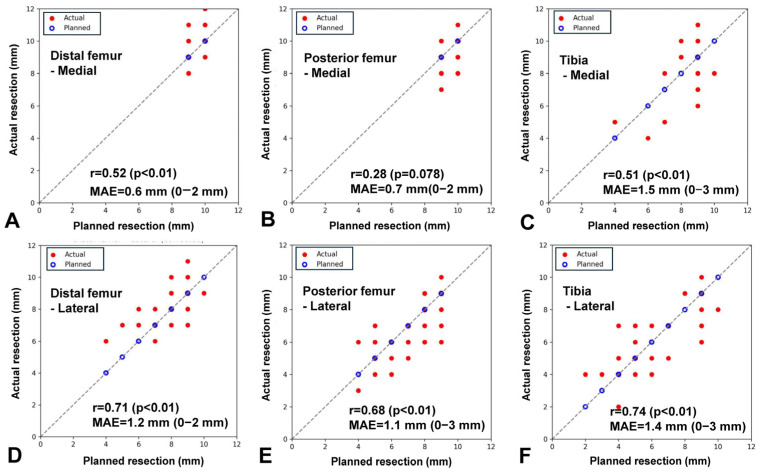
Agreement between planned and actual bone resection thickness. Scatter plots comparing planned and actual bone resection thickness for (**A**) distal femur–medial, (**B**) posterior femur–medial, (**C**) tibia–medial, (**D**) distal femur–lateral, (**E**) posterior femur–lateral, and (**F**) tibia–lateral. The dashed line represents the line of identity. Correlation coefficients (r), *p*-values, and mean absolute error (MAE) demonstrate the surgical accuracy of contralateral knee–referenced, caliper-verified uKA.

All surgeries were performed in a standard manner by a single surgeon (one of the authors) using a caliper-verified uKA technique through a standard medial parapatellar approach. The distal femur was cut using an intramedullary cutting guide, and the tibial cut was made following an extramedullary guide. Cartilage thickness was assessed using preoperative bilateral knee MRI, with measurements obtained at the distal femur, posterior femur, and tibia. When there was a bony intrusion, we derived patient-specific cartilage offset from MRI of the contralateral, non-OA knee (segmented hyaline cartilage thickness at the distal and posterior femoral condyles and the tibial plateau). We mapped those offsets to the operative side [26] (Figure 1). The calculated cartilage thickness was applied using discrete calibration bars (1, 2, or 3 mm) placed between the cutting guide and the worn compartment so that the saw cut reproduced the cartilage-compensated target, and each resection was verified with calipers (Figure 2). The accuracy and reproducibility of the caliper-verified technique have been validated in previous studies [2,27,28]. Caliper measurement of femoral and tibial resection thickness during KA TKA enables precise restoration of bone resection, with reported mean absolute errors of 0.5 to 1.5 mm and high interobserver reliability. In addition, standardized intraoperative gap assessment using tensor devices under controlled distraction forces has also demonstrated good reproducibility in evaluating soft-tissue balance. In the present study, we confirmed the technical accuracy of this approach by comparing planned and achieved bone resection thickness. Moderate-to-strong correlations were observed across all compartments, with mean absolute errors ranging from 0.6 to 1.5 mm (Figure 3), supporting the precision of contralateral-referenced, caliper-verified uKA in this cohort. Measurement of the medial and lateral gaps in full extension and 90° flexion was performed using a tensor device (B Braun-Aesculap, Tuttlingen, Germany) and scaled-force forceps (B Braun-Aesculap, Tuttlingen, Germany) as previously described [29]. The tensor device has a base plate placed on the resected tibial surface and two independent top plates placed on the medial and lateral resected femoral undersurfaces. In addition, it can be distracted and maintained separately, with two independent scales connected to each plate that indicate the distance from the base plate to the top plate. The forceps include a scale (range: 50–250 N) that indicates the force applied to the tensor device. The tip of the base plate and each of the top plates were placed perpendicular to both the resected tibial and femoral surfaces, and the tensor device was pushed into the joint as deeply as possible to prevent the top plates from bending. During measurement of the extension gap, the thigh and lower leg were supported by assistants to maintain full extension, and 90° flexion of the knee was maintained during the 90° flexion gap measurement. During each measurement, an investigator (one of the authors) applied a 200-N force, as indicated by the forceps scale, to both the medial and lateral top plates, and the measurement in millimeters, as read from the tensor device scale, was recorded.

**Figure 4:**
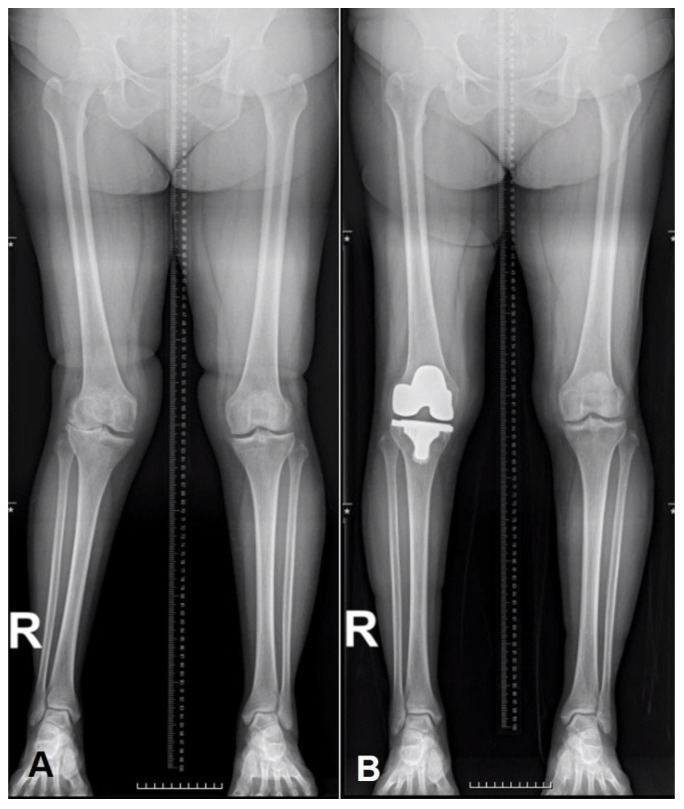
Preoperative and postoperative full-length standing radiographs. Preoperative standing anteroposterior radiograph demonstrating coronal alignment in a knee with lateral compartment OA (**A**). Postoperative radiograph showing restoration of coronal alignment after TKA using contralateral knee–referenced uKA, while maintaining alignment symmetry with the contralateral knee (**B**).

Surgical outcomes included resected bone thickness at the distal femur, posterior femur, and tibia, as well as medial and lateral gaps in full extension and at 90° of flexion. Radiographic outcomes included mechanical hip–knee–ankle angle (mHKA), medial proximal tibial angle (MPTA), lateral distal femoral angle (LDFA), arithmetic hip–knee–ankle angle (aHKA), joint line obliquity (JLO), and Coronal Plane Alignment of the Knee (CPAK) classification. Radiographic measurements were performed on standardized long-leg standing radiographs. To further characterize individual coronal alignment phenotypes, the coronal alignment of each knee was classified using the CPAK classification, a radiographic system that describes knee alignment based on constitutional limb alignment and joint line orientation. The CPAK classification incorporates two independent parameters: aHKA and JLO. The aHKA was calculated as the difference between the MPTA and LDFA (aHKA = MPTA − LDFA), representing constitutional coronal limb alignment independent of joint space narrowing. JLO was assessed using the arithmetic joint line obliquity (aJLO), calculated as aJLO = 90° − (MPTA + LDFA)/2, which reflects the degree of joint line orientation relative to the ground. Based on the combination of aHKA (varus, neutral, valgus) and joint line orientation (apex distal, neutral, or apex proximal), the CPAK system categorizes knees into nine distinct coronal alignment phenotypes, providing a comprehensive framework for describing knee alignment characteristics [30,31,32]. Clinical outcomes were assessed using PROs, including Pain VAS, New Knee Society Score (KSS) satisfaction, EQ-5D, and FJS, collected preoperatively and at final follow-up (Figure 4) [33].

All outcomes were compared between preoperative and postoperative states and between operated and contralateral knees. Continuous variables were analyzed using paired t-tests, and categorical variables, including CPAK classification, were compared using Fisher’s exact test when appropriate. The relationship between planned and actual bone resection thickness was evaluated using Pearson correlation analysis. All computations were performed with standard software (SPSS version 25.0 for Windows; IBM Corp., Armonk, NY, USA) with statistical significance set at *p* < 0.05. A sample size analysis indicated that at least 34 knees would be required to achieve 80% power to detect clinically meaningful differences at an alpha level of 0.05; therefore, the final cohort of 40 patients was considered adequate.

To assess the clinical relevance of changes in PROs, improvements were interpreted in relation to previously reported minimal clinically important differences (MCID). Based on previously published TKA literature, MCID thresholds are approximately 2 points for Pain VAS (0–10 scale), 5 points for the New KSS satisfaction subscale (0–40 scale), 0.08–0.12 for the EQ-5D index (corresponding to approximately 1 point in the summed score format), and 10–12 points for the FJS [34,35,36].

## 3. Results

Medial osteochondral resection thickness was consistently greater than lateral resection thickness at the distal femur, posterior femur, and tibia (all *p* < 0.01). In addition, the medial gaps were smaller than the lateral gaps in both full extension and 90° flexion (both *p* < 0.01). Despite these differences, the gap balance was well maintained, with a medial–lateral gap difference ≤ 2 mm observed in 95% of knees in full extension and in 70% at 90° flexion (Table 2).

**Figure 5:**
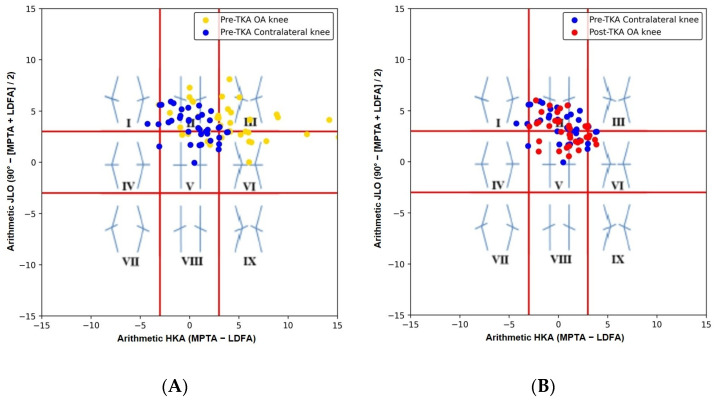
Coronal plane alignment distribution based on the CPAK concept. Preoperative OA knees (yellow) and contralateral knees (blue) plotted using arithmetic HKA angle (HKA; MPTA − LDFA) and arithmetic JLO (JLO; 90° − [MPTA + LDFA]/2) (**A**). Comparison of preoperative contralateral knees (blue) and postoperative OA knees after caliper-verified uKA (red) (**B**). Red reference lines indicate ±3° thresholds for HKA and JLO, defining the nine CPAK alignment zones.

Preoperatively, OA knees demonstrated a coronal alignment pattern distinct from that of the contralateral non-OA knees. On the CPAK scatter plots, the distribution of pre-TKA OA knees differed from that of the contralateral knees (Figure 5A), with a corresponding difference in CPAK classification (*p* < 0.01). In contrast, after uKA, post-TKA OA knees showed a CPAK distribution similar to that of the contralateral knees (Figure 5B), with no difference in CPAK classification between the two groups (*p* = 0.802).

All postoperative PROs, including Pain VAS, New KSS satisfaction, EQ-5D, and FJS, improved significantly compared with preoperative values (*p* < 0.01 for all comparisons). In this study, mean pain VAS improved from 7.3 to 1.5 (Δ = 5.8), the New KSS satisfaction from 12.1 to 31.2 (Δ = 19.1), the EQ-5D from 9.8 to 6.2 (Δ = 3.6), and the FJS from 19.7 to 96.1 (Δ = 76.4). All observed changes substantially exceeded the established MCID thresholds, indicating not only statistical significance but also clear clinical relevance from the patient’s perspective. At 2 years postoperatively, no differences were observed in any PRO between the operated knees and the contralateral non-OA knees (all *p* > 0.1) (Table 3).

## 4. Discussion

The most important finding of this study was that, when appropriate anatomic references are used, caliper-verified uKA in lateral compartment OA can reliably restore knee stability and CPAK phenotype comparable to the contralateral non-OA knee, while achieving equivalent patient-reported outcomes. These findings indicate that concerns regarding coronal malalignment, postoperative soft-tissue instability, or worse PROs in valgus knees treated with calipered uKA may be overstated.

The results of this study indicate that caliper-verified uKA in valgus OA resulted in asymmetric bone resection patterns with well-maintained intraoperative gap balance. Medial bone resections were approximately 2 mm greater than lateral bone resections at the distal femur, posterior femur, and tibia, with 95% of knees showing a medial-lateral gap difference ≤ 2 mm in full extension. This indicates that, unlike MA TKA, which relies on bone cuts perpendicular to the mechanical axis followed by extensive soft-tissue balancing, caliper-verified uKA can achieve acceptable intraoperative balance primarily through appropriate, anatomy-based bone resection alone [37,38]. The present findings are consistent with previous studies demonstrating that caliper-verified uKA can achieve reliable gap balance through precise bone resection alone, even in challenging valgus morphologies [9,22,39]. These results suggest that caliper-verified uKA can accommodate the inherent asymmetry of valgus knees while maintaining acceptable intraoperative stability, supporting the feasibility of anatomy-based reconstruction without reliance on extensive ligament release.

The data of this study suggest that caliper-verified uKA resulted in a postoperative CPAK phenotype of the operated knees that was comparable to that of the contralateral non-OA knees. In this study, a preoperative disparity in CPAK phenotype distribution between valgus OA and contralateral knees (*p* < 0.01) was no longer observed after caliper-verified uKA, with postoperative distributions showing no difference compared with the contralateral knee (*p* = 0.802). This finding supports that, unlike MA TKA, which often shifts knees toward a limited set of coronal alignment categories, caliper-verified uKA preserves coronal alignment characteristics and JLO even in valgus knees, extending observations previously reported mainly in varus populations [40,41,42]. Restoration of the CPAK phenotype may also have important biomechanical implications, as preservation of native coronal alignment and JLO may influence soft-tissue laxity and knee kinematics, potentially contributing to improved joint awareness. Many surgeons accustomed to MA TKA harbor concerns that applying caliper-verified uKA in valgus knees may lead to excessive postoperative valgus alignment. However, our study demonstrates that caliper-verified uKA in valgus OA yields a postoperative CPAK phenotype similar to that of the contralateral non-OA knee, indicating that when guided by appropriate anatomic references and precise bone resection, caliper-verified uKA inherently restores the patient’s individualized coronal alignment phenotype regardless of varus or valgus morphology. Nevertheless, whether restoration of individualized CPAK phenotypes offers advantages in implant longevity or functional outcomes over MA TKA, which predominantly converges toward CPAK type V, remains unclear and requires further investigation. Beyond descriptive alignment analysis, restoration of a CPAK phenotype consistent with the contralateral knee may have implications for implant longevity. Restoring individualized alignment and JLO could promote more physiologic load distribution and balanced lower limb biomechanics, potentially influencing polyethylene wear patterns and the risk of late revision. Although long-term survivorship data are not available in the present study, further investigation is warranted to determine whether phenotype restoration translates into improved implant durability.

Our findings suggest that caliper-verified uKA in valgus OA resulted in improvement with postoperative scores comparable to those of the contralateral non-OA knees. In this study, Pain VAS, satisfaction, EQ-5D, and FJS improved significantly after surgery (all *p* < 0.01), with scores comparable to those of the contralateral non-OA knee at 2 years postoperatively (all *p* > 0.1). Several studies have reported that residual valgus alignment or soft-tissue imbalance after TKA may negatively affect patient satisfaction and functional outcomes, particularly in valgus knees [18,19,20]. However, other studies have shown that KA TKA in valgus OA yields PROs that are not inferior to those in non-valgus knees and are associated with high patient satisfaction [9,22,39,43]. Our findings extend prior observations by demonstrating that uKA can achieve postoperative PROs comparable to those of the patient’s contralateral knee, notwithstanding longstanding concerns about valgus alignment and medial laxity.

MA TKA remains the most widely adopted alignment strategy in valgus knees, largely due to concerns about excessive valgus positioning and postoperative instability [18]. The MA approach aims to achieve neutral alignment and often requires lateral soft-tissue release to correct deformity, which may alter the native JLO and converge knees toward a narrower range of coronal alignment phenotypes, frequently corresponding to CPAK type V. In contrast, uKA seeks to restore patient-specific anatomy by reproducing individualized coronal alignment and JLO. In the present study, contralateral-referenced uKA restored individualized alignment phenotypes and achieved favorable short-term PROs without clinically relevant instability. These findings suggest that, in carefully selected valgus knees, uKA may serve as a feasible alternative to MA TKA. However, direct comparative studies are required to determine whether restoration of native alignment results in differences in long-term implant survivorship or functional outcomes.

Several limitations of this study should be noted. First, the predominance of Asian women in the study cohort may limit generalizability; however, this distribution is consistent with the well-documented demographic characteristics of patients with advanced knee OA in Korea [44,45]. Second, this study was a retrospective study, which should be considered when interpreting the findings. Lateral compartment OA with a preserved contralateral knee represents an uncommon clinical presentation, and a retrospective approach allowed identification of consecutive cases meeting strict inclusion criteria over a defined period, thereby ensuring cohort homogeneity and adequate follow-up. However, retrospective studies are inherently subject to potential selection bias, unmeasured confounding variables, and information bias. Although all procedures were performed by a single experienced KA surgeon using a standardized protocol to minimize variability, residual confounding cannot be excluded. Therefore, future prospective or comparative studies are needed to validate these findings. Third, the sample size was relatively small. Valgus knee OA itself is less common than varus OA. The specific presentation of a valgus knee with advanced OA (KL grade 4), accompanied by a contralateral knee with minimal degenerative change (KL grade ≤ 2), is exceedingly rare, which may limit the generalizability of our findings to broader valgus knee populations [46]. Consequently, some statistical findings, particularly small differences in radiographic parameters such as MPTA and LDFA, should be interpreted with caution, as the risk of type II error cannot be excluded; statistically significant differences may not necessarily reflect clinically meaningful effects. Fourth, the follow-up period was limited to a mean of approximately 2 years. Although postoperative alignment and clinical outcomes were satisfactory in the short term, a small degree of residual valgus alignment was observed in some cases, and its potential influence on long-term implant survivorship or late failure remains unknown. Long-term follow-up is required to determine whether these alignment characteristics affect durability over time. Fifth, this study did not include a comparison group treated with alternative alignment strategies, such as MA or FA. The absence of a direct comparison substantially limits the ability to determine whether contralateral-referenced uKA is superior to other alignment techniques in this specific patient population. Direct comparative studies are warranted to further clarify the relative benefits and potential trade-offs of uKA in valgus OA. Sixth, the high FJS observed in this study should be interpreted with caution. The strict inclusion criteria, including patients with isolated lateral compartment OA and a relatively preserved contralateral knee, may have resulted in a highly selected cohort with favorable biomechanical characteristics, potentially contributing to elevated PROs. Furthermore, the homogeneous study population and short-term follow-up raise the possibility of a ceiling effect of the FJS, which may limit the ability to detect differences in joint awareness. Finally, all procedures were performed without robotic assistance, relying on manual instrumentation and caliper-based verification, which may introduce measurement error. However, all procedures were performed by a single experienced surgeon using a caliper-verified technique, and both the accuracy of caliper-based bone resection and the reliability of intraoperative gap measurement have been previously validated in the literature [2,29,47,48]. Importantly, the caliper-verified approach reflects the surgical environment encountered by the majority of surgeons worldwide who do not have robotic systems, thereby enhancing the practical relevance of the present findings. Despite these limitations, the present results suggest that caliper-verified uKA can be a feasible option for treating lateral compartment valgus osteoarthritis, a rare and clinically challenging subset, when guided by appropriate anatomic references.

## 5. Conclusions

In valgus knees with lateral compartment OA, caliper-verified uKA can be safely and effectively performed when guided by accurate anatomic references and precise preoperative planning. Under these conditions, appropriate bone resection allows restoration of soft-tissue balance and CPAK phenotype comparable to the contralateral non-OA knee, with PROs of similar magnitude. These findings challenge the prevailing concern that uKA in valgus OA knees inevitably results in excessive valgus alignment, instability, or inferior functional outcomes, and support caliper-verified uKA as a feasible surgical option in carefully selected cases. However, the present findings reflect short-term clinical and radiological outcomes, and further studies with long-term follow-up are required to confirm the long-term safety and durability of this approach.

## Figures and Tables

**Table 1:** Preoperative characteristics *.

Demographic Factor	(*n* = 40)		
Age	71.4 (8.3)		
Gender (women) §	32 (80)		
Weight (kg)	61.1 (9.9)		
Height (cm)	156.7 (7.1)		
BMI (kg/m^2^)	24.8 (3.1)		
**Radiographic factor**			
	Operated	Contralateral	*p*-value
mHKA (°) ⁋	7.0 (7.2)	−0.9 (2.6)	<0.01
MPTA (°)	88.1 (3.0)	86.7 (2.0)	<0.01
LDFA (°)	84.1 (2.2)	86.3 (1.4)	<0.01
**CPAK**			
aHKA †	4.0 (4.0)	0.4 (2.1)	<0.01
JLO ‡	3.9 (1.7)	3.5 (1.4)	0.041
Classification §			<0.01
I	0	1 (3)	
II	13 (33)	23 (57)	
III	10 (25)	0	
IV	0	0	
V	6 (15)	14 (35)	
VI	11 (27)	2 (5)	

BMI, body mass index; mHKA, mechanical hip knee ankle angle; MPTA, medial proximal tibial angle; LDFA, lateral distal femoral angle; CPAK, coronal plane alignment of the knee; aHKA, arithmetic angle; JLO, joint line obliquity. * Data are presented as means (standard deviation); § Data are presented as number of patients (percentage); ⁋ Negative values indicate varus. † aHKA = MPTA − LDFA; ‡ JLO = 90 − (MPTA + LDFA)/2.

**Table 2:** Surgical and postoperative radiographic outcomes *.

Surgical Outcomes				
	Medial	Lateral		*p*-Value
Resected osteochondral fragment (mm)				
Distal femur	9.7 (0.9)	7.9 (1.3)		<0.01
Posterior femur	9.1 (0.8)	6.3 (1.5)		<0.01
Tibia	8.0 (1.9)	6.0 (1.8)		<0.01
**Gap (mm)**				
Full extension	11.9 (2.1)	12.6 (2.0)		<0.01
90° flexion	12.8 (2.2)	14.9 (1.9)		<0.01
Gap difference §	|Medial − Lateral|			
	≤2 mm		>2 mm	
Full extension	38 (95)		2 (5)	
90° flexion	28 (70)		12 (30)	
**Radiographic outcomes**				
	Operated	Contralateral		*p*-value
mHKA (°) †	1.8 (2.4)	−0.9 (2.6)		<0.01
MPTA (°)	87.3 (1.9)	86.7 (2.0)		<0.01
LDFA (°)	86.6 (1.4)	86.3 (1.4)		0.026
CPAK distribution §				0.802
I	0	1 (3)		
II	20 (50)	23 (57)		
V	18 (45)	14 (35)		
VI	2 (5)	2 (5)		

mHKA, mechanical hip–knee–ankle angle; MPTA, medial proximal tibial angle; LDFA, lateral distal femoral angle; CPAK, coronal plane alignment of the knee. * Data are presented as means (standard deviation); § Data are presented as the number of patients (percentage); † Negative values indicate varus.

**Table 3:** Postoperative changes of patient-reported outcomes *.

*n* = 40	Operated Knee			Post-TKA Status		
	Preoperative	Postoperative	*p*-Value	Operated	Contralateral	*p*-Value
Pain VAS	7.3 (0.8)	1.5 (1.6)	<0.01	1.5 (1.6)	1.3 (1.6)	0.739
Satisfaction	12.1 (4.6)	31.2 (10.1)	<0.01	31.2 (10.1)	31.4 (9.0)	0.944
EQ-5D	9.8 (1.1)	6.2 (1.8)	<0.01	6.2 (1.8)	6.0 (1.6)	0.719
FJS	19.7 (6.2)	96.1 (4.3)	<0.01	96.1 (4.3)	95.8 (5.1)	0.852

TKA, total knee arthroplasty; VAS, visual analogue scale; EQ-5D, EuroQol 5 Dimensions. FJS, forgotten joint score. * Data are presented as mean (standard deviation).

## Data Availability

The data that support the findings of this study are available from the corresponding author upon reasonable request.

## Abbreviations

The following abbreviations are used in this manuscript:

uKA	Unrestricted Kinematic Alignment
TKA	Total Knee Arthroplasty
OA	Osteoarthritis
CPAK	Coronal Plane Alignment of the Knee
PROs	Patient-Reported Outcomes
KA	Kinematic Alignment
MA	Mechanical Alignment
rKA	Restricted Kinematic Alignment
FA	Functional Alignment
FJS	Forgotten Joint Score
KL	Kellgren-Lawrence
mHKA	mechanical Hip–Knee–Ankle angle
MPTA	Medial Proximal Tibial Angle
LDFA	Lateral Distal Femoral Angle
